# Streamlining experiment design in cognitive hearing science using OpenSesame

**DOI:** 10.3758/s13428-022-01886-5

**Published:** 2022-07-06

**Authors:** Eleonora Sulas, Pierre-Yves Hasan, Yue Zhang, François Patou

**Affiliations:** 1 Oticon Medical, Vallauris, France; 2 Oticon Medical, Smørum, Denmark

**Keywords:** OpenSesame, Cognitive hearing science, Experiment design, Experiment building

## Abstract

Auditory science increasingly builds on concepts and testing paradigms originated in behavioral psychology and cognitive neuroscience – an evolution of which the resulting discipline is now known as cognitive hearing science. Experimental cognitive hearing science paradigms call for hybrid cognitive and psychobehavioral tests such as those relating the attentional system, working memory, and executive functioning to low-level auditory acuity or speech intelligibility. Building complex multi-stimuli experiments can rapidly become time-consuming and error-prone. Platform-based experiment design can help streamline the implementation of cognitive hearing science experimental paradigms, promote the standardization of experiment design practices, and ensure reliability and control. Here, we introduce a set of features for the open-source python-based OpenSesame platform that allows the rapid implementation of custom behavioral and cognitive hearing science tests, including complex multichannel audio stimuli while interfacing with various synchronous inputs/outputs. Our integration includes advanced audio playback capabilities with multiple loudspeakers, an adaptive procedure, compatibility with standard I/Os and their synchronization through implementation of the Lab Streaming Layer protocol. We exemplify the capabilities of this extended OpenSesame platform with an implementation of the three-alternative forced choice amplitude modulation detection test and discuss reliability and performance. The new features are available free of charge from GitHub: https://github.com/elus-om/BRM_OMEXP.

## Introduction

The last three decades have brought significant advances of our understanding of how cognitive processes tie in with auditory and language processing (Pisoni, factors, & implants, 2000; Dryden, Allen, Henshaw, & Heinrich, 2017; Rönnberg et al., 2016; Pisoni, Kronenberger, Chandramouli, & Conway, 2016; Hillyer et al., 2019). On the one hand, models of speech understanding and language acquisition have incorporated “top-down” mechanisms to account for intelligibility of speech even when the acoustic input signal is noisy or poorly represented at the peripheral level (Mosnier et al., 2015; Thomson, Auduong, Miller, & Gurgel, 2017; Völter, Götze, Dazert, Falkenstein, & Thomas, 2018; Kim, Lim, Kong, & Choi, 2018; Edwards, 2016; Peelle & effort, 2018). On the other hand, investigations of the reverse causal arrow of how hearing loss does influence cognition have also multiplied, looking for instance at the consequences of early life auditory deprivation on neurocognitive development or at the growing amount of evidence linking mid-life hearing loss to cognitive decline and dementia in older adults. (Livingston et al., 2017; Livingston et al., 2020; Thomson et al., 2017; Lin et al., 2011).

Research spanning auditory perception and cognitive science generally shows potential for translation to clinical applications. Interventions targeting hearing loss, e.g., hearing aids and cochlear implants may show differential benefits depending on a user’s cognitive abilities and therefore open up room for personalization strategies based on cognitive profiling (Moberly & Reed, 2019; Smith, Pisoni, & Kronenberger, 2019; Tamati, Ray, Vasil, Pisoni, & Moberly, 2020; Völter et al., 2021; Kestens, Degeest, & Keppler, 2021; Moberly, Lewis, Vasil, Ray, & Tamati, 2021). For instance, speech inputs provided by cochlear implants (CI) are heavily degraded (Zeng, 2004; Henry, Turner, Behrens, & resolution, 2005). CI users more than normal hearing individuals need to rely both on auditory acuity (i.e., bottom-up process) and their linguistic-cognitive abilities (i.e., top-down process) to understand speech. However, it remains unclear how the two processes interact to explain the great variability in speech understanding performance across CI users (Hillyer et al., 2019; Tamati et al., 2020; Moberly et al., 2021; Völter et al., 2021). Understanding this interaction could help us better predict which candidate will benefit poorly from a CI, or to propose a more individualized fine-tuning and rehabilitation. To address such questions, experiments typically need to test both bottom-up (for instance, amplitude modulation detection, spectral-temporally modulated ripple test (Aronoff & Landsberger, 2013), spectro-temporal ripple for investigating process or effectiveness (Archer-Boyd, Southwell, Deeks, Turner, & Carlyon, 2018), etc.) and top-down skills (for instance, working memory, verbal learning, executive control, verbal fluency, attention, linguistic skills etc. (Pisoni et al., 2016; Pisoni, Kronenberger, Harris, & Moberly, 2017; Moberly et al., 2021)), to try and better account for outcome performances on measures of sentence recognition or listening effort for instance.

Despite the current fervor, cognitive hearing science (CHS) comes with its set of challenges (Arlinger, Lunner, Lyxell, & Kathleen Pichora-Fuller, 2009; Pisoni, 2021). One is the consequence of extending the set of auditory science concepts and methods to incorporate those of behavioral psychology and cognitive neuroscience. For experimentalists, the resulting large CHS research space entails the use of multi-modality stimuli (i.e. audio, visual, tactile) and their synchronous coupling with objective neuroimaging and neurophysiological measurement techniques such as pupillometry (for the assessment of listening effort), functional near infra-red spectroscopy (fNIRS), and electroencephalograpy (EEG) for instance to explore selective attention (Miles et al., 2017; Alhanbali, Dawes, Millman, & Munro, 2019; Seifi Ala et al., 2020; Fiedler et al., 2021; Zekveld, Koelewijn, & Kramer, 2018; Wiggins, Anderson, Kitterick, & Hartley, 2016; Mackersie & Cones, 2011).

Experimental research paradigms in auditory science are still generally custom-built, and labor-intensive. Indeed, the design and implementation of auditory experiments commonly requires for instance the a priori design and recording of sound material in a sound studio, the pre-mixing of various tracks in specialized software, followed by the implementation of the experimental workflow with the control of playback timing, the specification of the sequence and order of audio playback, etc. The implementation of this experimental workflow is generally carried out with programmatic tools such as MATLAB or Python. Conversely, behavior psychology researchers and cognitive neuroscientists have shown earlier on greater interest in *platform-based* solutions to build their experimental workflows, i.e., relying on prefabricated logical building blocks, in an attempt to streamline their implementation effort – that is to standardize the control of timing for their various stimuli, to standardize compatibility with available neuroimaging and neurophysiology equipment, and to simplify the specification of experimental logic (e.g., number of experimental blocks, loops, stop conditions, etc.). Until today, CHS researchers had to rely on the custom-built approach in order to ensure the availability of all the advanced audio features required for their experiments thereby preventing them from harnessing the benefits of platform-based experiment building for the behavioral and cognitive aspects of their research. Here, we present a platform-based experiment builder offering the audio control features commonly sought by auditory scientists. We build on the open-source OpenSesame platform (Mathôt, Schreij, & Theeuwes, 2012) to streamline the implementation of behavioral and cognitive hearing science experiments that include multi-channel audio stimuli while interfacing with various synchronous I/Os through the Lab Streaming Layer protocol.

### Cognitive and behavioral experiment builders

Mathôt et al. give an extensive account of the main solutions available for cognitive science experiment building (Mathôt et al., 2012). We summarize and elaborate on their findings with CHS applications in mind. Of the available experiment-building platforms, few are based on a commonly used scripting language, a feature that means that the specification of highly specific elements of computation or logic that are not readily available is not easily accessible. On the other hand, only PsychoPy, E-Prime, and Presentation make extensive use of a graphical user interface and of “drag-and-drop” mechanisms to enhance usability, a feature that can help streamlining experiment building for many researchers who are not proficient with coding.

Since then, Peirce et al. introduced PsychoPy2 building on PsychoPy (Peirce et al., 2019). One of the important additions to the original PsychoPy package is the option of using two kinds of interfaces, one for scripting in Python, and the other for constructing experiments graphically and the addition of the ioHub system for asynchronous control of various pieces of hardware, mainly conceived for eye-tracking purposes.

Mathôt et al. introduced OpenSesame, an experiment-building platform for cognitive science with extensive functionality, written in Python, compatible with PsychoPy, allowing scripting and available as open-source software under the GPLv3 license. OpenSesame is a graphical experiment builder, made of a collection of so-called “plugins” that are to be understood as modular functional blocks. Plugins are available for displaying text, images, or videos on screen, collecting user-input, recording data from different hardware peripherals, and even offering basic functionality for playing back audio stimuli, all as part of a user-centric laboratory experiment. OpenSesame proposes a little-code user interface, which can be particularly useful for non-technical users who do not know scripting/coding but are keen to design their own experimental paradigms. OpenSesame will also appeal to code-skilled users, who will be able to specify advanced functionality using Python. OpenSesame offers to run implemented experiments using a simplified version of its interface or “runner” version.

## An OpenSesame-based platform for hearing science

We introduce a set of plugins for OpenSesame that enable the implementation of behavioral and cognitive tasks involving advanced audio playback. For clarity, we refer to the OpenSesame platform enhanced with these plugins as the Oticon Medical Experiment Platform (OMEXP). OMEXP is meant to facilitate the implementation and execution of behavioral and cognitive hearing science experiments and, by this means to support researchers and experimentalists looking to address cognitive hearing science questions. OMEXP was implemented in Python on a 64-bit Windows machine. The current version of OMEXP was built on OpenSesame version 3.2.7, available at (Mathôt & et al. 2019).

### Features

The implementation of advanced audio capabilities in OpenSesame required the implementation of a low-level audio mixing library and a set of plugins harnessing it. The *Audio Mixer* plugin allows the playback of a virtually unlimited number of monaural .wav audio files (in practice limited by the memory of the computer running OpenSesame) on an unlimited number of audio channels each set at a specific sound pressure level (SPL), with a specific timing, and all rendered through virtually any currently available sound card. A *Calibration* plugin enables a plug-and-play audio calibration per channel provided that the operator running the test possesses a soundmeter to provide a ground truth measurement of the audio playback SPL. The *Calibration* plugin is bundled with white noise but also allows using a different audio file to run the calibration with. If an input track has more than one channel then they are summed into one. Moreover, a set of plugins was implemented to allow the recording of synchronous input data streams from various devices provided they implement the Lab Streaming Layer (LSL) protocol, (Kothe & et al. 2019a). LSL is a system enabling the unified collection of time-series measurements in research experiments. LSL handles networking, time-synchronization, (near-) real-time access to these data streams as well as the centralized collection, viewing, and disk recording of the data carried by these streams (Kothe & et al. 2019b). The list of plugins added to OpenSesame to constitute OMEXP are summarized below and described in detail in the following section. The new plugins are available in GitHub (https://github.com/elus-om/BRM_OMEXP) and they can be pip-installed inside OpenSesame following the installation instructions.

- Audio Mixer,
- Calibration,
- LSL start,
- LSL message,
- LSL stop,
- Adaptive init,
- Adaptive next,

### Plugins

#### Audio mixer

The *Calibration* and *Audio Mixer* plugins extend the default audio features provided by OpenSesame. The OpenSesame *Sampler* plugin only supports two channels and a linear volume control while the OMEXP *Audio Mixer* can theoretically support any number of outputs as well as a control of the volume in decibel (dB), which can also be linked to a dB SPL value through the use of the *Calibration* plugin. Both the *Audio Mixer* and *Calibration* plugins rely on the core libmixer.py library file built with the Rtmixer (Geier & et al. 2021), Numpy (Harris et al., 2020), Soundfile (Bechtold & et al. 2019), and Resampy (McFee & et al. 2019) libraries.

The libmixer.py library file allows opening and processing of audio files of interest through a chosen audio output device and audio API pair. Through the *Audio Mixer* plugin parameters’ pane, the experiment designer can specify the path to the .wav files to be read, the playback level for each .wav file, specified in dB with respect to a reference, the channels through which each .wav file should be played, and whether to notify other plugins of the beginning or end of the overall mixed signal with a specified message over LSL.

The names of the audio files to be played must be added to the OpenSesame file pool. During the *prepare* phase of the experiment execution, the .wav files are first read using the *Soundfile* library and then resampled to the chosen output device default sampling rate using the *Resampy* module. The output is then converted to a mono signal with a root mean square (RMS) value set to unit value. Using the *NumPy* library, each unit-level RMS signal is then scaled and added to different columns of a vector. The *Audio Mixer* is based on the use of the Mixer class provided by the *Rtmixer* module, which takes in that vector of values and a corresponding mapping vector linking each audio signal to a specified audio output channel. The combination of Python, Windows, and PortAudio (Bencina & Burk, 2001) (called by *rtmixer*) generates some jitter or timing imprecision on playback, down to the granularity of the audio blocks output by the machine. The magnitude of this jitter is an important consideration for some CHS experiments, for instance for low-latency measurement experiments where stimuli are repeated and acquisition signals averaged such as in evoked response potential experiments. For such scenarios, the *Audio Mixer* plugin offers the experiment designer the option to fixing the audio buffer size to the minimum size (32 samples). Provided that the computer on which the *Audio Mixer* is running has enough RAM and CPU to cope with low-latency audio, this mode will limit jitter without any side effects such as audio glitches. For non-timing-critical experiments, the block size can be left variable, in which case it is managed by PortAudio. The audio is handled by a separate thread so as to allow synchronous, concurrent visual stimuli to be presented by OpenSesame.

#### Audio calibration

While the *Audio Mixer* allows the experiment designer to specify a playback level, the value that is specified is only used to scale the signal and does not have a corresponding physical value in dB SPL by default. The *Calibration* plugin remedies that by playing a calibration signal (by default a white noise) and allowing the operator to adjust the baseline level specified in software to correspond to the ground truth SPL measured in the room. The calibration parameters are saved as experiment variables and applied to the *Audio Mixer*’s signals vector right before playing the audio stimuli ensuring a playback at the specified output SPL.

#### LSL plugins

The Lab Streaming Layer protocol provides an abstraction layer for the network-based real-time acquisition of time-series data coming from peripheral applications also implementing LSL. LSL relies on a core *liblsl* library (Kothe & et al. 2019b), which can be handled in Python using the *pylsl* Python package (Kothe, 2019).

LSL is based on the use of Stream Outlet, Stream Inlet, and Resolver classes. Stream Outlet makes the time-series data streams available on the LSL network. Hardware manufacturers interested in making the data their equipment generates available on the LSL network need to implement an *App* (using LSL terminology) that overwrites Stream Outlet. The Resolver collects and interprets the different Stream Outlets. It queries based on the name, type, or some other value field to list the available Stream Outlets on the LSL network that match the request. Once the query has been answered, the Resolver creates the relevant Stream Inlet to receive data from the specified Streams. Multiple-devices Stream Outlets can be acquired at the same time and their time-series data are mutually synchronized. The built-in time synchronization of the different time-series data streams acquired through LSL is based on the use of time stamps and correction routines, all relying on the high-resolution clock of the computer running the LSL applications. The resulting synchronous multiple time-series data streams can be saved into a single XDF (eXtended Data Format) file to serve further data processing or analysis.

Here, three OpenSesame plugins were developed together with an LSLsession class independent from OpenSesame based on the *pylsl* (Kothe, 2019) and *LieSL* (Guggenberger, 2019) libraries. *LieSL* provides a number of tools to manage LSL streams. Once the targeted Stream Outlets are resolved and an output file name is specified (together with a target directory), *LieSL* is able to start the recording of multiple Outlet Streams and to save the stream data and meta-data in an XDF file, until the recording is stopped. The LSLsession relies both on the *pylsl* and *LieSL* libraries to resolve the available streams and manage the recordings.

The *LSL start* plugin allows the initialization of the LSLsession, create the Outlet Stream named *Logger*, if selected, and start the recording of selected data streams. The created LSLsession instance is then stored as an OpenSesame experiment variable. The *LSL message* allows the annotation of the timestamped data streams at a specific moment during acquisition with a custom-defined message push through the *Logger* Outlet stream. The *LSL message* plugin gets the available LSLsession object from the experiment variable pool, and it pushes the markers to the *Logger* stream. The *LSL stop* plugin simply stops the recording. It retrieves the LSLsession object from the experiment variable pool, previously created and stops the current recording.

#### Adaptive procedures

Adaptive procedures are useful in many psychophysics experiments. For instance, when estimating speech reception threshold (SRT) for noisy speech, a non-adaptive procedure will require testing participants at every single signal-to-noise ratio (SNR) step, in order to fit psychometric functions to the participants’ performance. Then, the estimation of a certain threshold (e.g., SRT50% or SRT85%) will be calculated from that psychometric fitting. This non-adaptive approach is not only time-consuming, but also not efficient, because only the specific threshold estimates are needed. In comparison, an adaptive approach allows for an effective search of the threshold point without sweeping across the entire range of the input parameter. An adaptive procedure will instead change the input stimuli depending on the test participant’s previous responses until it converges to its target value, or according to predetermined rules/methods. This adaptive approach is more efficient, and is greatly used in auditory research, both in lab and clinic (Leek, 2001; Kollmeier, Gilkey, & Sieben, 1988; Brand & Kollmeier, 2002). The transformed up-down procedure has been the most widely used method for adaptive procedure both in research and clinical application (Levitt, 1971). OpenSesame by default already contains an adaptive procedure implementing the QUEST algorithm (Watson & Pelli, 1983), a Bayesian method requiring prior knowledge to estimate thresholds. For many psychoacoustics experiments relevant to CHS research, an adaptive up-down procedure such as the one introduced in (Levitt, 1971) is needed. This method uses sequences of positive or negative responses to modify a physical value of the stimuli, e.g., its intensity. We implement this up-down procedure through two new OpenSesame plugins: *Adaptive Init* and *Adaptive next*.

The *Adaptive Init* plugin allows the specification of the defining parameters of the up-down procedure: the step sizes, the number of reversals or trials after which the step-size changes may occur, the variable holding the tracked value, and its starting value. Implementation of both reversals counts and trial counts are possible. We provide a ready-made implementation of stop-by-reversals as it is fairly cumbersome (i.e., one needs to calculate psychometric convergence, count the reversals during the experiments, and keep track of the reversals, etc.). In comparison, stop-by-trials is easier to specify (i.e., the default *count trial* variable already provides all the information needed). The experimenter still has the freedom to override the *Adaptive* plugin to stop the tracking by adding a break condition statement in the *Loop* plugin. The *Adaptive next* plugin takes as parameter the name of the variable holding the result of the task (correct or incorrect), and computes the next value of the tracked variable, defined earlier, based on the history of answers.

## Platform validation

In order to both illustrate the capabilities offered by the features introduced by OMEXP and to characterize the platform overall performance (in terms of timing specifically), we present the implementation of a three-alternative forced choice (3-AFC) amplitude modulation detection test (AMDT). The aim of the test is to identify the acoustic modulation depth threshold at which a test participant can identify the amplitude modulation (AM) tone sound stimulus when presented with two un-modulated tones and one AM tone, with a 50% accuracy. This test is used to establish the temporal processing abilities of the listener and of the signal processing. 3-AFC AMDT has been implemented using different tools including MATLAB (De Ruiter, Debruyne, Chenault, Francart, & Brokx, 2015), LabVIEW (Dimitrijevic et al., 2016), or combination of platforms, e.g., separating the stimulus generation from the behavioral test response parts of the test such as in (Han & Dimitrijevic, 2020).

We present an OMEXP implementation of the 3-AFC AMDT in the next section followed by a series of validation steps of both logic and timing performance. The 3-AFC AMDT experiment file and the data used for the validation are shared in Mendeley data (Sulas, Hasan, Zhang, & Patou, 2020).

### 3-AFC AMDT implementation

**Fig. 1:**
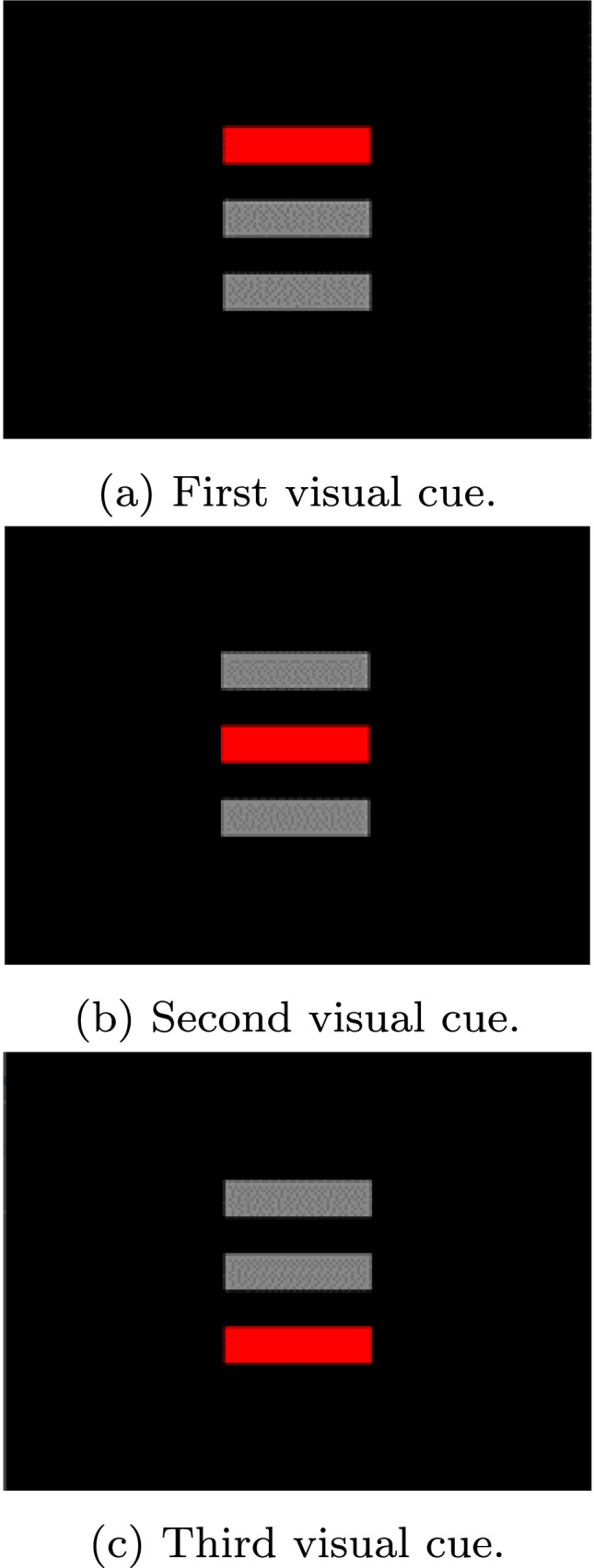
Visual cues used for the 3-AFC-AMDT. Three sound stimuli are sequentially played and each stimulus is associated to one of the *red rectangles* displayed on a screen in front of the participant

**Fig. 2:**
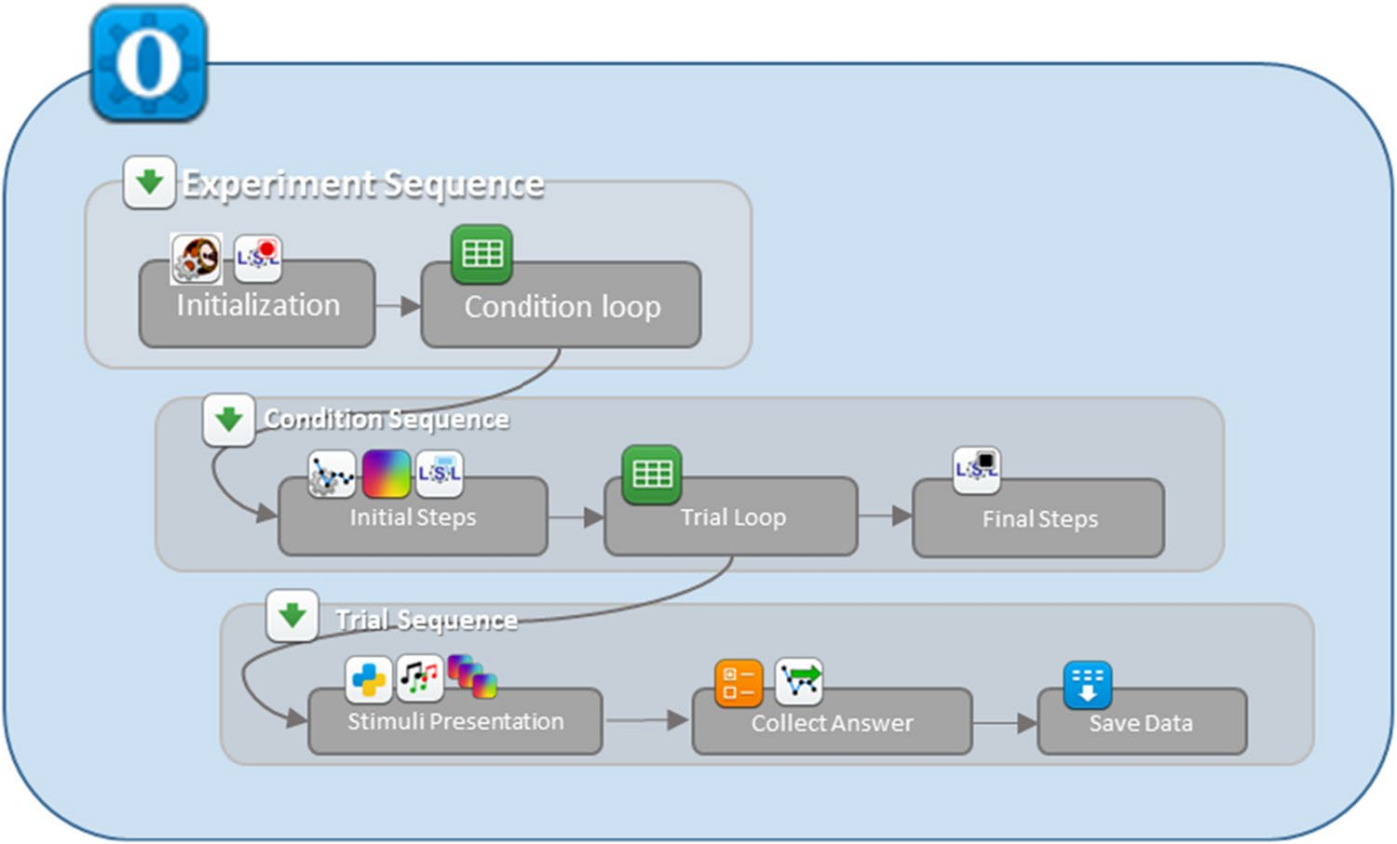
3AFC AMDT logic translated to the experiment structure

The 3-AFC AMDT means to determine the minimum acoustic modulation depth a test participant can identify in an amplitude modulated (AM) tone acoustic stimulus. Three sound stimuli (two amplitude-flat tones and one AM tone (the “target”) are played sequentially, in random order, to the test participant. Each sound is associated to a red rectangle displayed on a screen in front of the participant (see Fig. 1). The test participant should click or touch the area on the screen with which she believes the target sound to be associated. The 3-down-1-up adaptive procedure tracks a variable, in this example [depth]. [depth] should be decremented one step after three correct answers and incremented after one wrong answer, using one unit step size for the first reversal and an adjusted step size for the following reversals. This routine is repeated for a fixed number of iterations or until a number of successful trials or reversals has been reached. The final value of [depth] corresponds to the threshold we are looking for. The 3-AFC AMDT can be specified as an OMEXP experiment structure as shown in Fig. 2 by using *Loops* and *Sequences*. The test starts by calibrating the sound setup and the LSL recording. The *Condition Loop* allows to run the *Condition sequence* which initializes the adaptive routine for each new condition and to run each condition. Sequentially, the *Trial Loop* will prepare and present the audio stimuli taking into account the tracked variable, collect the answer, and adjust the tracked variable based on the answer.

**Fig. 3:**
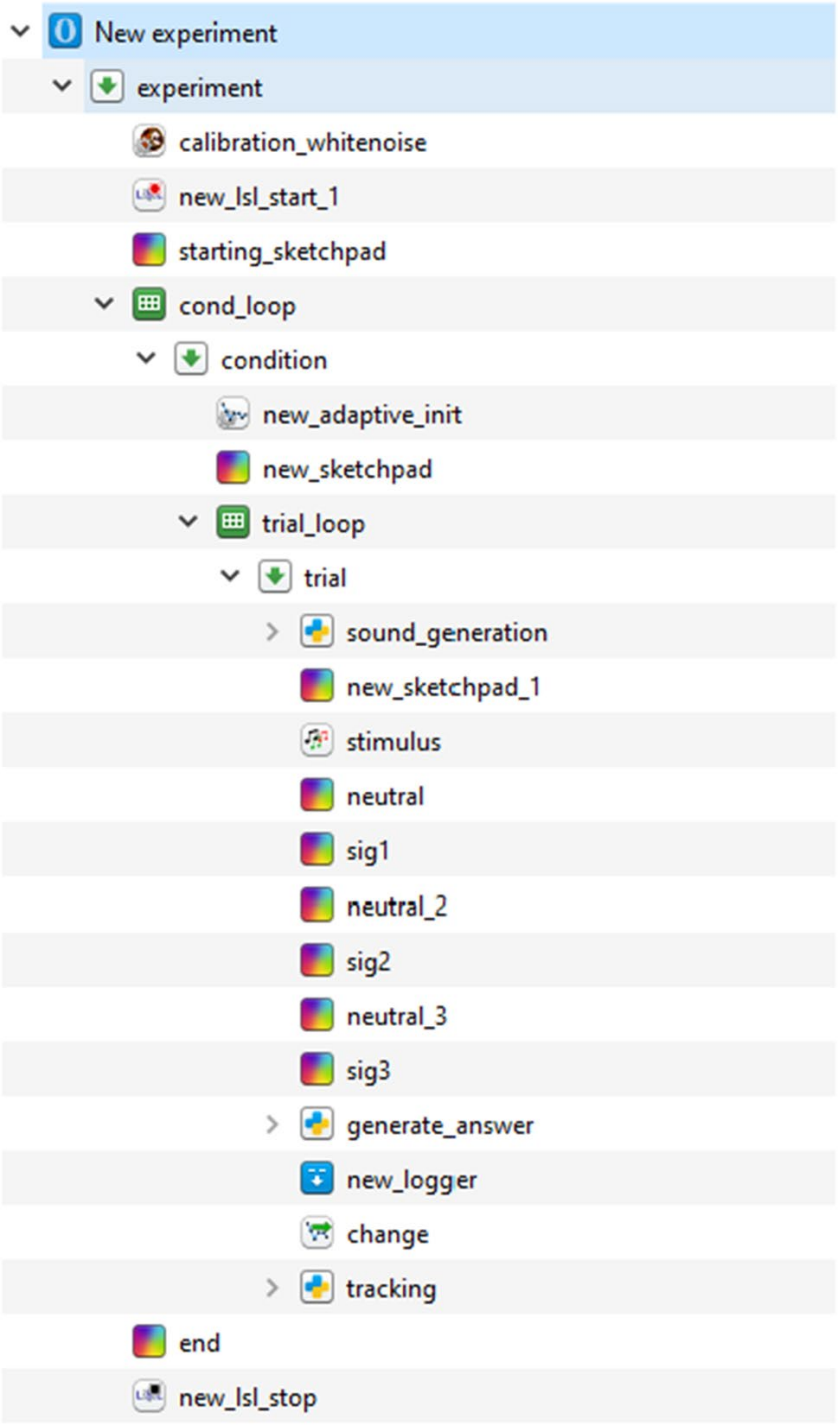
3AFC AMDT experiment sequence: it consists of a collection of actions (plugins) which will be run in a sequential order. *Calibration whitenoise, new lsl start 1, new adaptive init, stimulus* and *change* represent the new plugins introduced in this manuscript

The OMEXP implementation of the 3-AFC AMDT is illustrated in Fig. 3. It requires the *Calibration* plugin to be inserted before any audio playback plugin is called to ensure that the audio stimuli are delivered at the expected SPL. The *Audio Mixer* plugin is central to the experiment. *Sketchpads* plugins will allow the presentation of the red rectangles, and the *Adaptive next* plugin serves to change [depth], the depth of the modulation of the target signal (the *Adaptive Init* plugin must be used once in the initialization phase of the experiment). An *inline script* plugin is used to generate the audio stimuli before performing the main trial sequence is executed. OpenSesame gives the possibility to display instructions to the test participant using the *Sketchpads* plugin. This 3-AFC AMDT version was implemented for an automated validation purpose, therefore little to no instructions were included.

A condition *Loop* is used nests the overall main trial *Sequence* so that trials of modulation depth detection are run for different pure-tone and modulation frequencies (the conditions). Nested in the condition *Loop*, see Fig. 3, the main trial *Sequence* will be run several times by presenting an AM tone (at a specific frequency set by the *Condition loop*) in random order among the three audio stimuli of each trial. This is done thanks to the trial *Loop*.

**Fig. 4:**
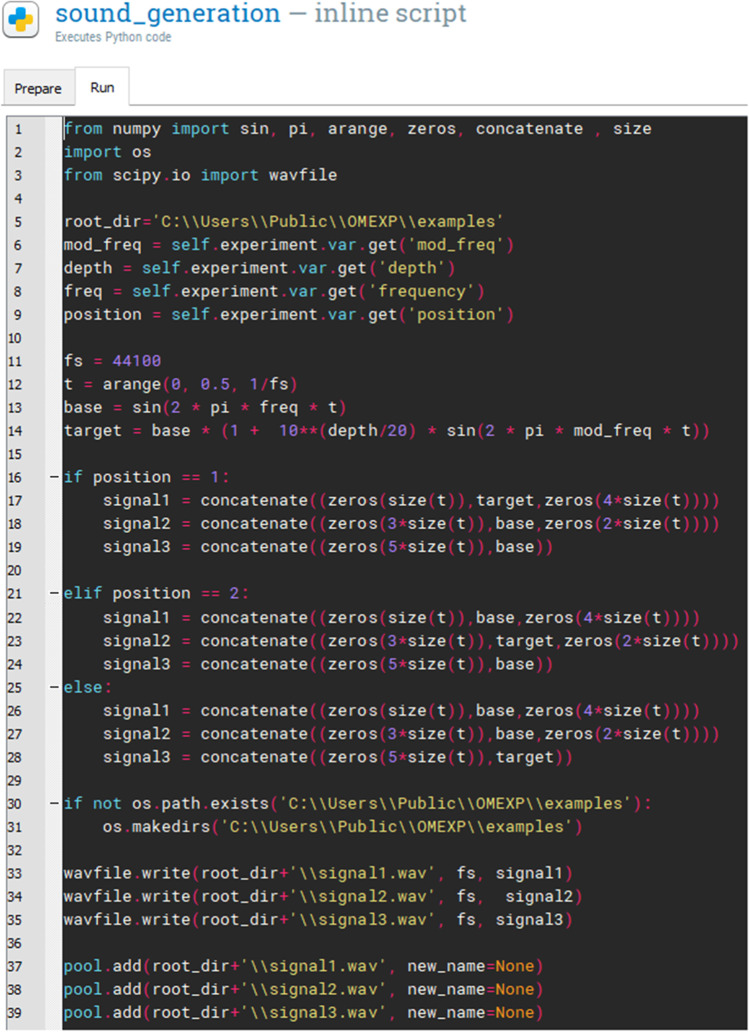
Inline script to generate the sound stimuli

The trial *Loop* randomizes the association between the target audio stimulus with one of the rectangular shape displayed on screen for each given trial, i.e., whether the AM tone will be related to the first, second, or third rectangular shape. For each iteration in the trial *Loop*, the audio stimuli are generated (at the given carrier frequency with a given modulation depth) using the *inline script* plugin. The Python script used to generate the audio stimuli is illustrated in Fig. 4.

**Fig. 5:**
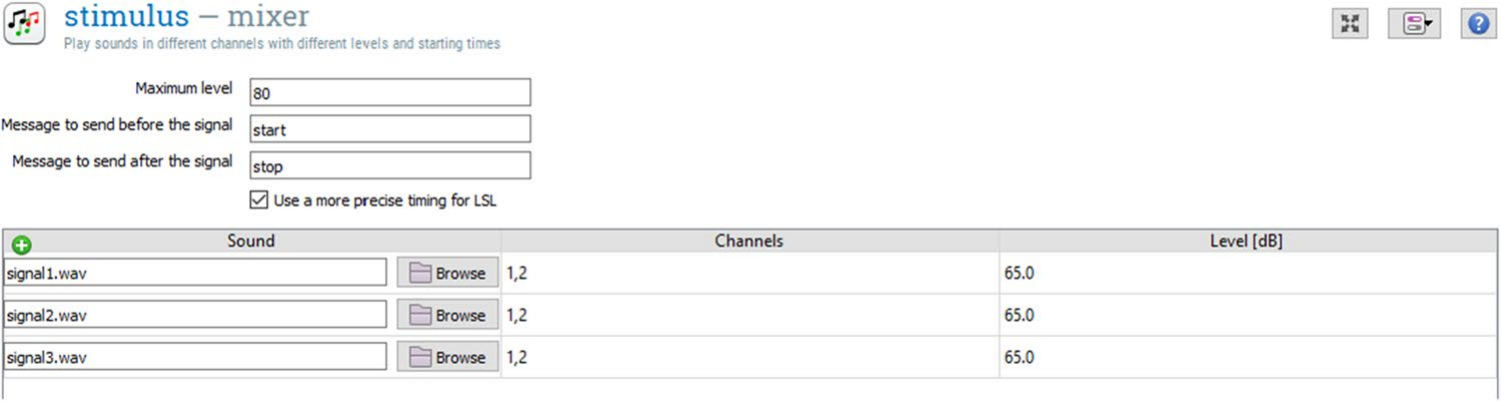
Mixer plugin

The *Audio Mixer* (Fig. 5) is then responsible for playing the three previously generated audio stimuli (signal1, signal2, and signal3 in Fig. 4). By timing the *Sketchpads*, signal1 can be played when the first red rectangle is shown (Fig. 1a), signal2 when the second red rectangle is presented (Fig. 1b), and signal3 when the third red rectangle is shown (Fig. 1c). Before the first rectangle appears and between the showing/hiding of the other two rectangles, a neutral sketchpad is shown. Signal1 starts to play 500 ms after the first neutral sketchpad is displayed, signal2 after 1500 ms (duration of the first neutral sketchpad + first rectangle + second neutral sketchpad), and signal3 after 2500 ms (duration of the first neutral sketchpad + first rectangle + second neutral sketchpad + second rectangle + third neutral sketchpad).

**Fig. 6:**
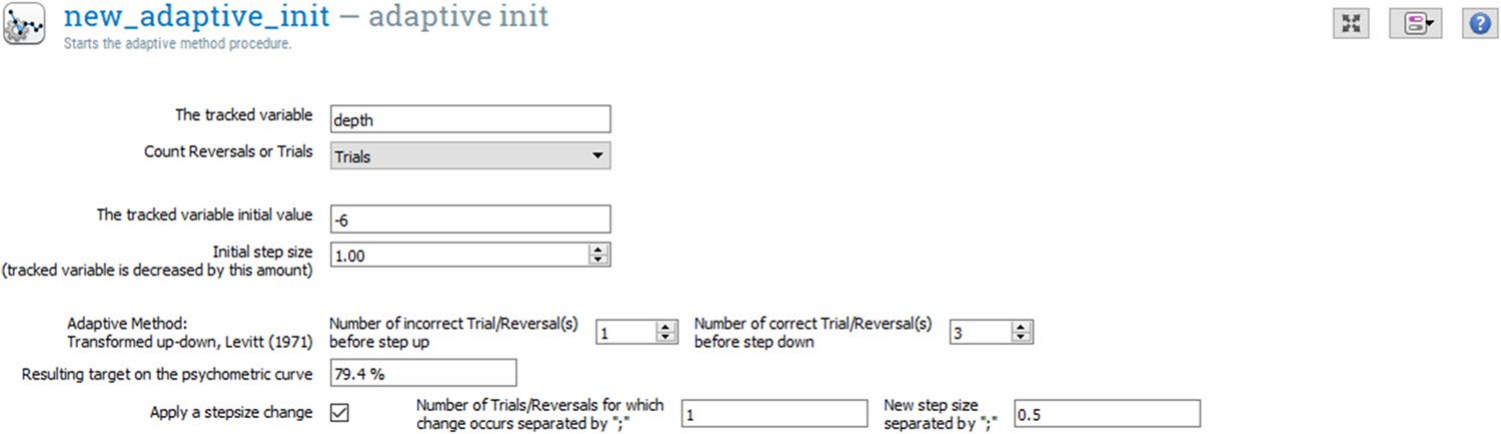
Adaptive plugin

The test participant response is then collected and the adaptive procedure can change the [depth] value, as shown in Fig. 6, where the initial value was set to 6. In this version of the 3-AFC AMDT experiment, we counted the trials and we used two different step sizes. The initial step size is set to 1 and, after it the step size changes to 0.5. However, it is possible to adjust the step size for each trial/reversal by writing a list of step sizes (in the field *New step size separated by ;*) and the number of reversals (in the field *Number of Trials/Reversals for which change occurs separated by ;*) separated with a semicolon, e.g., 1;0.5;0.2.

**Fig. 7:**
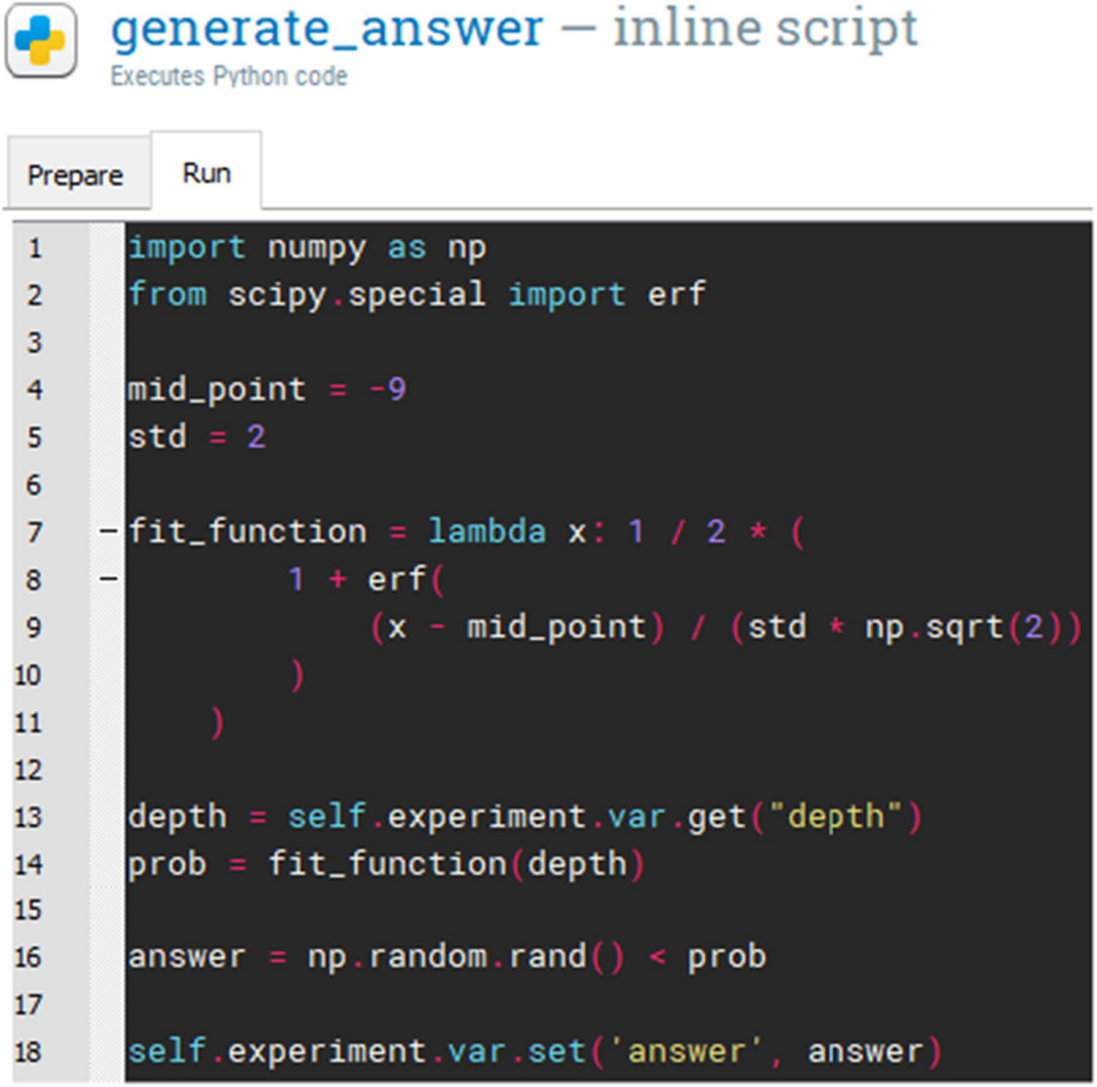
Automatic answer generated using an *inline script* plugin

**Fig. 8:**
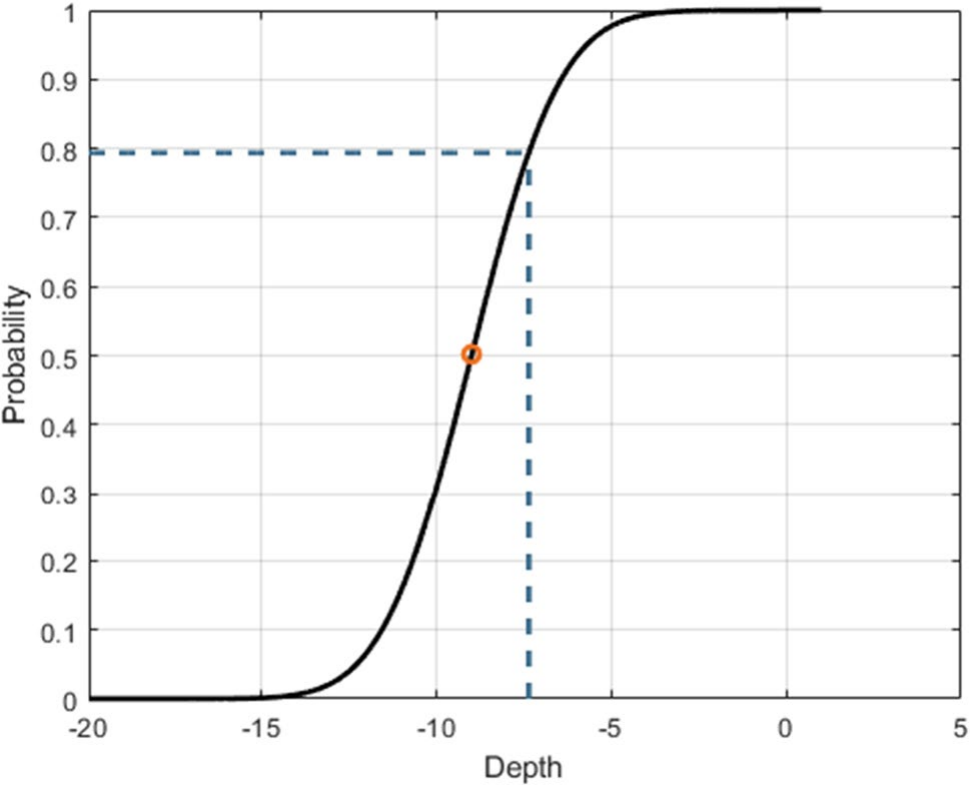
The psychometric function that was used to generate the synthetic response: the *orange circle* represents the midpoint of the curve and the *blue dashed lines* represent the expected convergence [depth] value our 3-down 1-up adaptive procedure

Here we simulate the test participant responses using an inline script (Fig. 7) implementing an inferential psychometric model (Fig. 8). The probability of correct response is modeled as a linear combination of predictors by means of a sigmoid link function according to the Eq. 1:

1 $$prob(depth) = {\frac{1}{2}\left[ 1 + erf\left( \frac{depth-midpoint}{std\sqrt{2}} \right) \right] }$$

where the midpoint is -9 dB and standard deviation (std) was set to 2, erf is the Gauss error function, a special (non-elementary) sigmoid function simulating a test participant with a -9 dB modulation detection threshold with a standard deviation of 2.

For the 3-down-1-up strategy that we are using here, we expect a probability of positive response at convergence *prob*(*depth*) = 0.794 (Levitt, 1971).

Figure 8 presents the psychometric function in black and the middle point in orange; the dashed line displays the expected probability for the 3-down-1-up procedure. Therefore, from the graph, we expect a modulation detection threshold converging towards a depth of -7.3622 dB.

The test participant answer is generated randomly for each iteration of the loop according to the function:

2 $$answer = rand < prob(depth)$$

where rand is a random number taken from a uniform distribution between 0 and 1.

The *Adaptive next* plugin uses the new variable [answer] to track the state of the adaptive procedure.

**Fig. 9:**
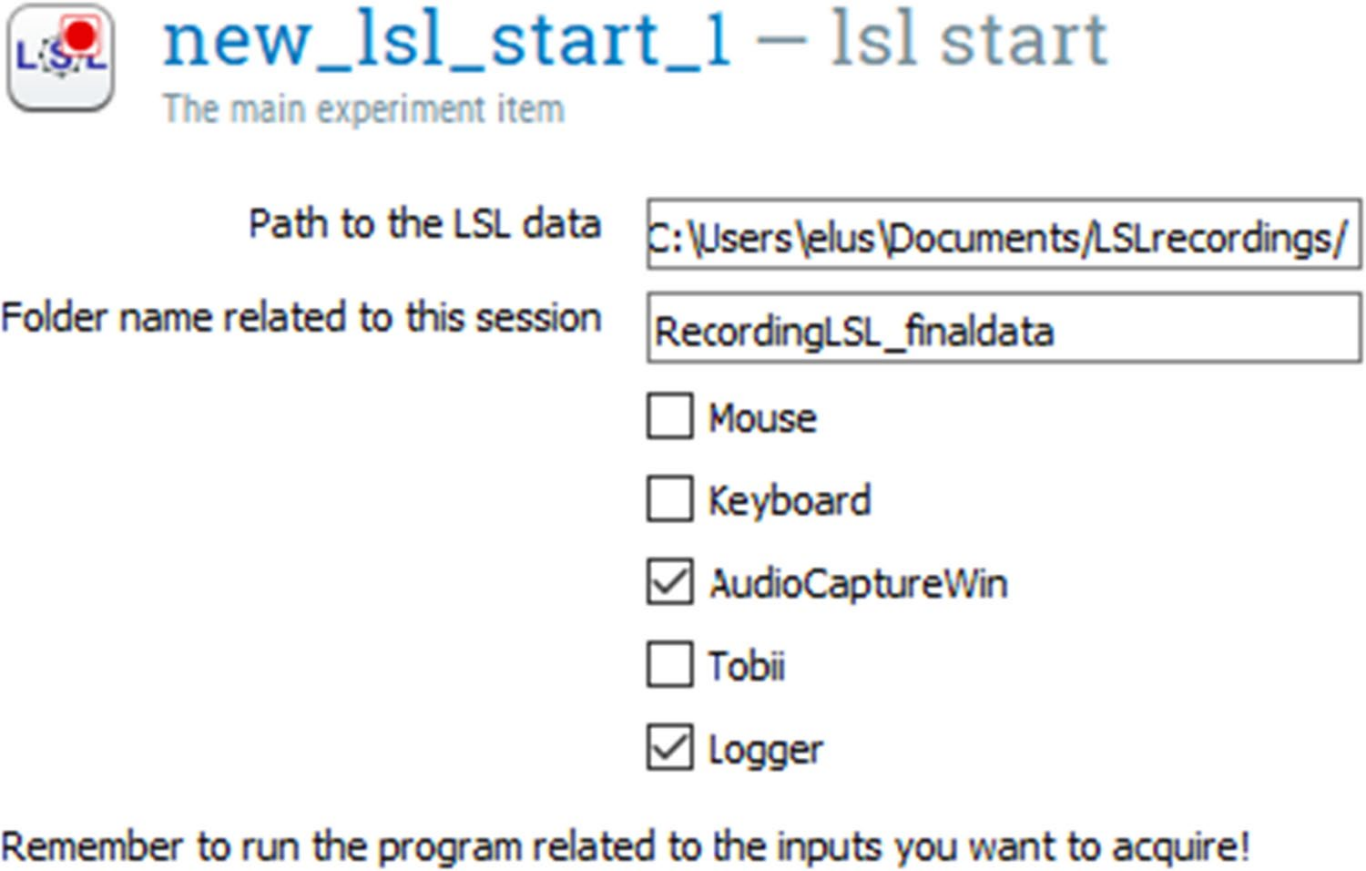
LSL start plugin

**Fig. 10:**
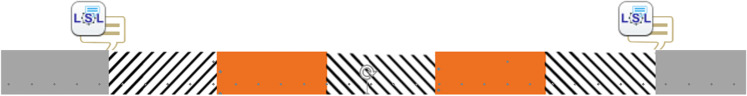
Visual representation of the sound stimuli and the specific moments at which the LSL messages are recorded. The *Audio Mixer* plugin plays three different audio stimuli sequentially, depicted as *striped boxes*. The *orange rectangles* represent the silent breaks between the three sound stimuli. For each audio stimulus, the *lsl message plugin* saves an LSL marker for the time in which the audio files starts and stops to be played

LSL streaming is initialized using the *LSL start* plugin (Fig. 9). In addition, LSL markers are created within the *Audio Mixer* plugin, at the beginning and at the end of the sequence of all three audio stimuli (message start and message end in Fig. 5). Figure 10 shows the time at which the three audio stimuli are played back and the LSL markers are saved.

### Platform behavior: validation

Because adaptive procedures are important buildings blocks of many psychophysics (including psychoacoustics) and hearing research experiments, we validated the behavior of the *Adaptive init* and *Adaptive next* plugins using a well-known adaptive methodology. We compute convergence estimation and estimation variability from a series of ten experiments with for each experiment the following four carrier frequency (f) and modulation frequency (mod_freq) combinations:

1. f = 400 Hz, mod_freq = 5;
2. f = 1000 Hz, mod_freq = 5;
3. f = 400 Hz, mod_freq = 10;
4. f = 1000 Hz, mod_freq = 10.

These four conditions were played for each simulated test participant in a random order three times. For each condition, the three possible target positions were played 12 times each. Therefore, 36 audio files have been generated and played for each of the four conditions three times, for a total of 144 audio files reproduced for each of the ten simulated participants.

The behavior of the adaptive procedure can be evaluated by looking into three parameters:

(a): convergence estimation, calculated as the converged values in the last six reversals of the smallest step size, (Levitt, 1971), as in Eq. 4:
  3 $$\begin{array}{@{}rcl@{}} depth_{mean}(j) = {\frac {1}{N}}\sum\limits_{i=1}^{N}depth(i,j) \end{array}$$
  where *N* is the number of trials across the last six reversals, and
  4 $$\begin{array}{@{}rcl@{}} convergence \quad estimation= {\frac{1}{M}} \cdot \\ \sum\limits_{j=1}^{M} \left( depth_{mean}(j) \right) \end{array}$$
  where *M* is number of simulated condition, that is equal to 4 conditions per simulated test participant (for a total of 4 ⋅ 10 observations). As explained before, looking at the graph in Fig. 8, we expect a convergence estimation as close as possible to − 7.3622 *dB*.
(b): convergence variability, calculated as the mean of the standard deviations of the converged values, calculated in the last six reversals of the smallest step size, across repetitions as in Eq. 6:
  5 $$\begin{array}{@{}rcl@{}} &&depth_{std}(j)= \sqrt{\frac{1}{N} \cdot} \\ &&\sqrt{\sum\limits_{i=1}^{N} \left( depth(i,j) - depth_{mean}(j) \right)^{2}} \end{array}$$
  where *N* is again the number of trials across the last six reversals, and
  6 $$\begin{array}{@{}rcl@{}} convergence \quad variability = {\frac{1}{M}} {\sum}_{j=1}^{M} \left( depth_{std}(j) \right) \end{array}$$
(c): estimation variability, calculated as the standard deviation from the converged values in each repetition to the target convergence value of − 7.3622 *dB*, as shown in Eq. 8:
  7 $$\begin{array}{@{}rcl@{}} diff_{depth}(j)= depth_{mean}(j) - (-7.3622) \end{array}$$
  8 $$\sqrt{{\frac1M\sum\limits_{j=1}^M\left({diff}_{depth}(j)-\overline{diff_{depth}}\right)}^2}$$

As mentioned earlier, the smallest stepsize is 0.5 dB.

**Fig. 11:**
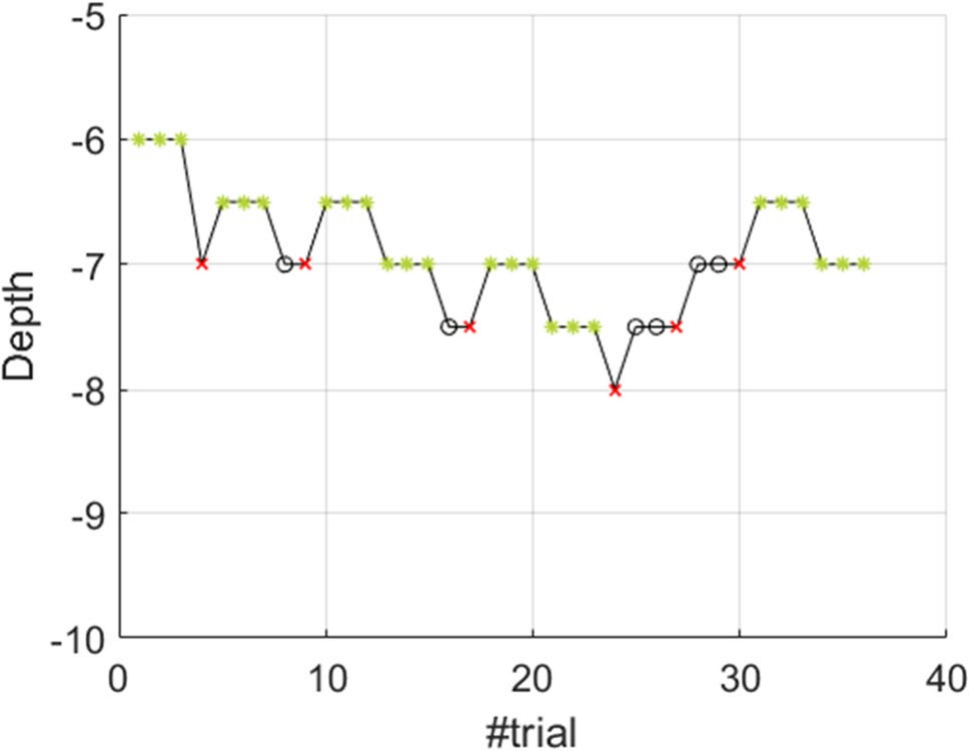
3-down-1-up procedure results for the first simulated test participant and for the first of the four conditions (frequency = 400 Hz and modulation frequency = 5 Hz)

Figure 11 presents the evolution of the [depth] variable value as a function of the trial index for the first simulated test participant for the first of the four conditions (frequency = 400 Hz and modulation frequency = 5 Hz). A total of 36 trials are run with triplets of audio stimuli repeated 12 times. The black circles represent a correct response (value= 1) and the red crosses represent a wrong answer. The green stars mark 3 consecutive correct answers, the point at which the [depth] value is incremented. Only one wrong answer triggers [depth] to decrement.

**Fig. 12:**
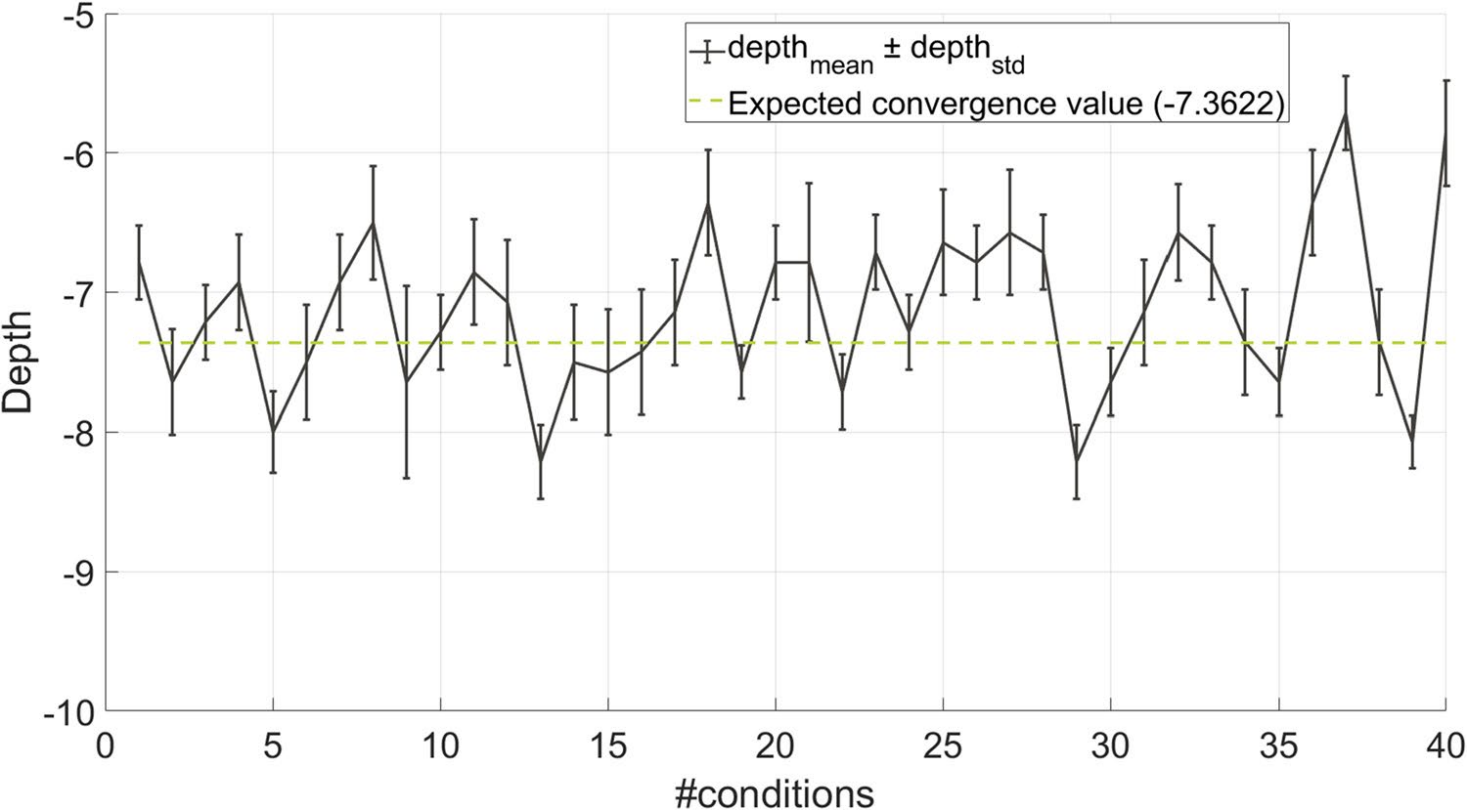
Platform behavior validation of the adaptive plugins. The *x*-axis represents the 40 tested conditions, four combinations of frequencies, and modulation frequencies for the ten synthetic subjects. The *y*-axis shows the depth value range. The *black line* shows the *depth*_*mean*_(*j*), where j goes from 1st to the 40th tested condition, and the *error bars* represents the *depth*_*std*_(*j*). The *dashed green line* gives the expected convergence value (=-7.3622 dB)

Our platform behavior validation shows that the convergence estimation and the convergence variability calculated for the last six reversals of the smallest step size across the four conditions for the ten simulated subjects, are -7.1214 dB ± 0.3431 dB, respectively. Figure 12 shows the evolution of the mean [depth] and its standard deviation represented as error bars for each of the 40 conditions (four combinations of frequencies and modulation frequencies per ten synthetic responses); the green line provides the expected convergence value (-7.3622 dB). The estimation variability is 0.5877 dB.

## Performance characterization

### LSL and *Audio Mixer* timing characterization setup

We aim to characterize the timing delays and jitter of OMEXP’s audio playback and LSL stream acquisition. We use the 3-AFC AMDT experiment skeleton presented above, and as the experiment runs, two LSL streams are acquired: the *Logger* and the audio stream through the stand-alone AudioCaptureWin application. An Outlet stream is created for the *Logger* and the samples are pushed into the stream by *LSLsession*.

AudioCaptureWin makes the computer’s microphone input available for recording over LSL. We close-loop our setup by wiring the output of the *Audio Mixer* output channel to the computer’s input microphone via an external USB audio interface (ESI Maya 44 USB+). The systematic error of our audio playback timings (corresponding to the average time difference between the onset of the audio stimulus as it is streamed out of the sound card versus the timestamp of the LSL sound start markers) can be construed as the average *latency* of our system and reflects LSL markers timing accuracy during audio recording. This systematic error is computed using Eq. 11:

9 $$diff (i,j) = xl(i,j) - xs(i,j)$$

where j stands for the index of the ten simulated test participants, i for the index of the audio stimulus (up to 48 sound triplets, so that *N* = 48), xl corresponds to the LSL sound start marker timestamp and xs corresponds to the timestamp of the onset of the audio stimulus at the output of the soundcard.

Because the *latency* is not constant, we must therefore repeat the measure and compute a statistical estimate of the mean time difference. This latency can be subtracted from all the measurements to make the *random error* or *jitter* associated with the system appear.

10 $$x_{mean}(j)={\frac {1}{N}}\sum\limits_{i=1}^{N}xl(i,j) - xs(i,j)$$

11 $$\begin{array}{@{}rcl@{}} &&random \quad error(i,j) \\ &&= diff(i,j)-x_{mean}(j) \end{array}$$

The standard error of the mean (SEM), corresponding to the fluctuations of the different latency estimates around their mean value, is expressed with equation:

12 $$SEM=\sqrt{{\frac1M\sum\limits_{j=1}^M\left(x_{mean}(j)-\overline{x_{mean}}\right)}^2}$$

where M is equal to the 10, which is the number of simulated test participants.

Various combinations of Windows 10 audio drivers (MME, DirectSound, and WASAPI) and ASIO settings (buffer sizes) were tested in an attempt to reduce both latency and jitter, eventually settling on the MME driver for which we present latency and jitter data in below.

### Audio playback performance

**Fig. 13:**
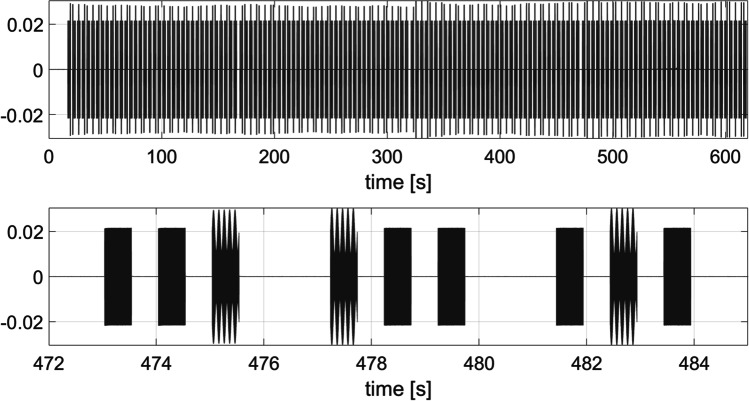
The acquired LSL sound stream is represented: *on the top*, the whole sound stream consisting of 144 AM tones is shown; *on the bottom*, the sounds related to one trial (3x3 audio files) are depicted

Figure 13 illustrates the sound streams acquisitions. In the first row, we can see the 144 sound tracks that have been played with by design 48 AM single tones and 96 non-modulated single tone audio stimuli. The second row shows the combination of the AM tone with the non-modulated tones, repeated three times, each time changing the location of the targeted audio source.

**Fig. 14:**
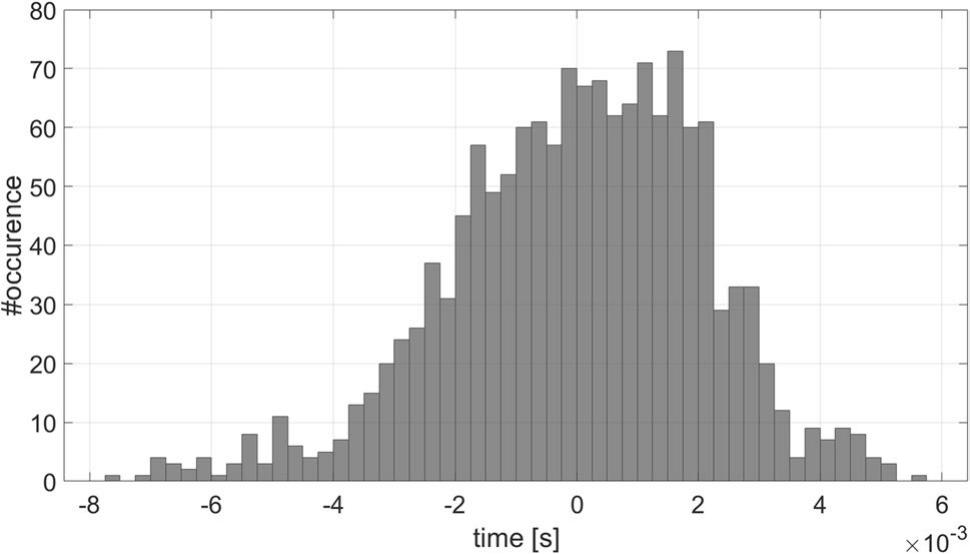
Random error distribution for the ten simulated subjects responses

Figure 14 shows the distribution of the random error, which corresponds to the deviations of the differences between the timestamps related to the physical sound starts and the LSL start markers, across the ten synthetic participants. The values of the first, second, and third quantiles are -1.3, 0.2, and 1.5 ms respectively. We considered the inter-quantile 75–25% range of 2.8 ms an acceptable level of jitter for many CHS experimental paradigms including those involving pupillometry or fNIRS monitoring. We concede that this range is too high for experiments looking at low latency physiological responses such as evoked response paradigms using EEG recording, as a jitter less than 1 ms is generally requested. The standard error of the mean, which corresponds to the fluctuations of the jitter estimate around its mean value, is 0.0035 s, demonstrating that the performances are repeatable and consistent.

## Discussion

The implementation of experimental paradigms in cognitive hearing science is generally complex and time-consuming. Building on the open-source OpenSesame platform, we propose a set of OpenSesame plugins that can help in streamlining the implementation of experiments involving advanced audio playback capabilities synchronously with visual stimuli and multimodal data acquisition from various physiological monitoring pieces of equipment. Implemented features include: advanced audio playback capabilities with multiple loudspeakers at various sound pressure levels and tracks, as well as control over timing, audio level calibration, a state-of-the-art adaptive procedure, and compatibility with standard inputs/outputs (I/Os) using LSL. These new plugins have been validated behaviorally including our adaptive routine plugin which we tested as part of an implementation of a 3-AFC ADMT. Importantly, we have characterized the timing performance of our audio playback module and demonstrated a very consistent sub-10-ms jitter which we are confident is sufficient for the general usability of OMEXP for many CHS experimental paradigms including those incorporating pupillometry or fNIRS. The time constant of pupil response to auditory stimuli for instance lies in the 100-ms range (Winn, Wendt, Koelewijn, & Kuchinsky, 2018). We must acknowledge that an average jitter in the 10-ms range does not allow for low-latency repeated objective measurements such as auditory evoked response potentials measured by EEG. Reducing this jitter will be the object of future work.

Setting aside this limitation, we could therefore envisage OMEXP to help with the homogenization of experiment design and data collection format in the cognitive hearing science, aiding researchers to better plan experiments and synthesize knowledge across experiments and sites (for instance, enabling meta-analysis and multi-centered studies) (Heinrich & Knight, 2020). We propose to start this homogenization effort with an OMEXP implementation of the widely used Hearing In Noise Test (HINT) (Nilsson, Soli, & Sullivan, 1994) adding concurrent pupillometry. Our OMEXP HINT implementation is included in the OMEXP default release and only requires adding and specifying the relevant audio files for the specific language of interest. It also includes the Sentence final Word Identification and Recall (Ng, Rudner, Lunner, Pedersen, & Rönnberg, 2013), with the same plug-and-play customizability with regards to the input audio files. Many other tests could possibly be shared and address research questions relating to listening effort, fatigue, cognitive capacity, and learning, etc. Further developments around the OpenSesame platform would be required to facilitate that process, including making our audio playback features web-compatible and enable the pooling of anonymous data across sites.

## Conclusions

Building on the open-source OpenSesame platform, OMEXP extends the features of the OpenSesame experiment builder so as to enable experiment implementation involving psychoacoustics and other hearing-related tasks coupled to multimodal objective data acquisition. OMEXP’s audio playback capabilities have been rigorously validated and will contribute to promoting the reliability and reproducibility of cognitive hearing experimental research.
